# Beobachtungsstudie zur Koinzidenz von Alzheimer-Erkrankung und idiopathischem Normaldruckhydrozephalus: Analyse von Koinzidenz, deren Einfluss auf das Ansprechen im Liquorablassversuch und zerebrovaskulärer Kopathologie

**DOI:** 10.1007/s00115-025-01808-8

**Published:** 2025-02-27

**Authors:** M. Beeke, C. Sauer, J. Petzold, S. Schneider, K. Frenzen, M. Donix, G. Reiß, M. Brandt, R. Haußmann

**Affiliations:** 1 Klinik und Poliklinik für Psychiatrie und Psychotherapie, Universitätsklinikum Carl Gustav Carus an der Technischen Universität Dresden, Fetscherstr. 74, 01307 Dresden, Deutschland; 2 Institut und Poliklinik für Diagnostische und Interventionelle Neuroradiologie, Universitätsklinikum Carl Gustav Carus und Medizinische Fakultät, Technische Universität Dresden, Fetscherstr. 74, 01307 Dresden, Deutschland; 3 Sächsisches Krankenhaus Arnsdorf, Akademisches Lehrkrankenhaus, der Technischen Universität Dresden, Hufelandstr. 15, 01477 Arnsdorf, Deutschland; 4 Klinik und Poliklinik für Neurochirurgie, Universitätsklinikum Carl Gustav Carus, an der Technischen Universität Dresden, Fetscherstr. 74, 01307 Dresden, Deutschland; 5 Klinik und Poliklinik für Neurologie, Universitätsklinikum Carl Gustav Carus, an der Technischen Universität Dresden, Fetscherstr. 74, 01307 Dresden, Deutschland; 6 DZNE, Deutsches Zentrum für Neurodegenerative Erkrankungen, Dresden, Deutschland; 7 Universitäts DemenzCentrum (UDC), Klinik und Poliklinik für Psychiatrie und Psychotherapie, Universitätsklinikum Carl Gustav Carus, an der Technischen Universität Dresden, Fetscherstr. 74, 01307 Dresden, Deutschland

**Keywords:** Evans-Index, Corpus-callosum-Winkel, Vaskuläre Kopathologie, Liquorablassversuch, Hakim-Trias, Evans index, Corpus callosum angle, Vascular copathology, Spinal drainage, Hakim’s triad

## Abstract

**Ziel der Arbeit:**

Analyse der Häufigkeit einer komorbiden Alzheimer-Erkrankung (AD) bei Patienten mit Verdacht auf idiopathischen Normaldruckhydrozephalus (iNPH) und deren Effekt auf das Ansprechen im Liquorablassversuch sowie Analyse der Häufigkeit einer vaskulären Kopathologie bei Patienten mit iNPH-Verdacht.

**Material und Methoden:**

Prospektiv beobachtende Analyse von Patienten mit iNPH-Verdacht, die sich im Rahmen der klinischen Routinediagnostik zwischen dem 01.07.2022 und dem 30.06.2023 einer leitliniengerechten NPH-Routinediagnostik inklusive Liquorablassversuch unterzogen. Die Rekrutierung erfolgte aus den Kliniken für Neurologie, Neurochirurgie und Psychiatrie des Universitätsklinikums Carl Gustav Carus in Dresden. NPH-typische Bildbefunde wurden anhand vorliegender MRT- bzw. CT-Schnittbilder erhoben. Relevante soziodemografische, klinische, kognitive und liquordiagnostische Parameter wurden mittels Aktendurchsicht erfasst. Die Patienten wurden hinsichtlich des Liquorbefundes gemäß ATN-Klassifikation kategorisiert.

**Ergebnisse:**

Im Beobachtungszeitraum wurden 33 Patienten (14 weiblich, 19 männlich, Durchschnittsalter 74,6 ± 8,1 Jahre) mit iNPH-Verdacht analysiert. 19 Patienten (57,6 %) wiesen eine komplette und 14 Patienten (42,4 %) eine inkomplette Hakim-Trias auf. Die Differenz des MoCA-Scores vor und nach Liquorablass unterschied zwischen Patienten mit und ohne Ansprechen im Liquorablass (F[1;22] = 5,725; *p* = 0,026). Es bestand ein Trend, dass Patienten mit pathologischem Corpus-callosum-Winkel und auffälligem Evans-Index (*p* = 0,052) sowie Patienten mit pathologischem Corpus-callosum-Winkel, Evans-Index und kompletter Hakim-Trias (*p* = 0,055) häufiger ansprechen. Der durchschnittliche Fazekas-Score betrug 1,7. Es bestand kein Zusammenhang zwischen Fazekas-Score und Ansprechen auf den Liquorablass. Insgesamt wurden bei 25 Patienten (75,8 %) Demenz- und Destruktionsmarker bestimmt. Gemäß ATN-Klassifikation wurden 20 Patienten (80 %) als A^+^T^−^, 3 (12,0 %) als A^+^T^+^ und 2 (8,0 %) als A^−^T^−^klassifiziert. A^+^T^+^- und A^+^T^−^-Patienten sprachen nicht häufiger auf den Liquorablass an (*p* = 0,600).

**Diskussion:**

Die kombinierte Bestimmung von CCW und Evans-Index sowie deren Kontextualisierung mit klinischen Charakteristika verbessern möglicherweise die prognostische Einschätzung eines Ansprechens im Liquorablassversuch. Insbesondere eine komorbide Amyloidpathologie, aber auch eine zerebrale Mikroangiopathie stellen häufige Kopathologien beim iNPH dar, wobei der Einfluss einer komorbiden Amyloidpathologie auf das Ansprechen im Liquorablassversuch noch ungeklärt ist.

## Abstract (en)

**Objective:**

Analysis of the frequency of comorbid Alzheimer’s disease in patients with suspected idiopathic normal pressure hydrocephalus (iNPH) and its effects on the response to cerebrospinal fluid (CSF) drainage as well as analysis of the frequency of a vascular copathology in patients with suspected iNPH.

**Material and methods:**

This was a prospective observational analysis of patients with suspected iNPH who underwent guideline-conform NPH routine diagnostics including CSF drainage during clinical routine diagnostics between 1 July 2022 and 30 June 2023. Patients were recruited via the departments of neurology, neurosurgery and psychiatry of the University Hospital Carl Gustav Carus in Dresden. Typical NPH imaging results were acquired from available magnetic resonance imaging (MRI) and computed tomography (CT) sectional images. Relevant sociodemographic, clinical, cognitive and CSF diagnostic parameters were acquired via patient chart review. The patients were categorized with respect to the CSF results according to the amyloid-tau-neurodegeneration (ATN) classification.

**Results:**

During the observational period 33 patients (14 female, 19 male, mean age 74.6 ± 8.1 years) with suspected iNPH were analyzed. Of the patients 19 (57.6%) had a complete and 14 (42.4%) an incomplete Hakim’s triad. The difference between the MoCA scores before and after CSF drainage varied between patients with and without a response to CSF drainage (F(1;22) = 5.725; *p* = 0.026). There was a trend that patients with a pathological corpus callosum angle and conspicuous Evans index (*p* = 0.052) as well as patients with a pathological corpus callosum angle, Evans index and complete clinical Hakim’s triad (*p* = 0.055) more frequently show a response. The mean Fazekas score was 1.7. There was no correlation between the Fazekas score and response to CSF drainage. In 25 patients (75.8%) biomarkers for dementia and neurodegeneration were detected. According to the ATN classification 20 patients (80%) were categorized as A^+^T^−^, 3 (12.0%) as A^+^T^+^ and 2 (8.0%) as A^−^T^−^. Patients classified as A^+^T^+^ and A^+^T^−^ did not respond more often to CSF drainage (*p* = 0.600).

**Conclusion:**

The combined determination of the corpus callosum angle and the Evans index as well es their contextualization with clinical characteristics can possibly improve the prognostic evaluation regarding response to CSF draínage. Especially a comorbid amyloid pathology and a cerebral microangiopathy represent frequent copathologies of iNPH but the influence on the response to CSF drainage remains to be elucidated.

## Hintergrund und Fragestellung

Beim idiopathischen Normaldruckhydrozephalus (iNPH) handelt es sich um eine progrediente Erkrankung mit kommunizierendem Hydrozephalus, die bildmorphologisch durch erweiterte Ventrikel bei normalem Liquoreröffnungsdruck (nicht mehr als 20 cm H_2_0) gekennzeichnet ist [1]. Der iNPH ist insbesondere im höheren Lebensalter häufig [2]. Bis zu 14 % aller Pflegeheimbewohner leiden beispielsweise an einem iNPH [3]. Das klinische Syndrom ist durch kognitive Defizite bis hin zu einem demenziellen Syndrom mit primär subkortikal-dysexekutivem Defizitprofil, eine Gangstörung und eine Dranginkontinenz charakterisiert, wobei die klassische Symptomtrias (Hakim-Trias) bei weniger als 60 % der Patienten vorliegt [4, 5]. Somit ist der iNPH insbesondere in der ätiologischen Differenzierung von Demenzsyndromen mit früher Gangstörung klinisch relevant [6]. Bei zeitgerechter Diagnose und Therapie durch eine ventrikuloperitoneale Shuntanlage ist die Symptomatik, in Abhängigkeit vom Vorliegen von Komorbiditäten, potenziell reversibel und darf deshalb nicht übersehen werden [2]. Gleichzeitig müssen diese Komorbiditäten aber auch erfasst werden, um wenig Erfolg versprechende operative Prozeduren zu vermeiden. Aufgrund des gegenwärtig fehlenden kohärenten Verständnisses zur Erkrankungsätiologie und Pathophysiologie ist die Diagnosestellung eines iNPH komplex [2].

Der wesentliche bildgebende Befund ist die Ventrikelerweiterung in Abwesenheit einer ausgeprägten kortikalen Atrophie [2, 6]. Das Missverhältnis zwischen Seitenventrikelerweiterung und vertexnah engem Kortexrelief („high convexity tightness“) stellt einen typischen Schnittbildbefund beim NPH dar. Das Vorliegen vertexnah enger Furchen, einhergehend mit erweiterten Sulci laterales bzw. fokal weiten äußeren Liquorräumen in anderer Lokalisation wird auch als DESH („disproportionately enlarged subarachnoid space hydrocephalus“) bezeichnet [7], wobei es sich aus diagnostischer Sicht um ein qualitatives Kriterium handelt [1, 2]. Der hinsichtlich der zugrunde liegenden Ätiologie unspezifische Evans-Index wird aus dem Verhältnis von maximaler Weite der Vorderhörner der Seitenventrikel auf einem axialen Schnittbild (cCT oder cMRT) und maximalem innerem Durchmesser des Schädels in derselben Schicht gebildet und gilt ab Werten > 0,3 als auffällig [1]. Der Corpus-callosum-Winkel (CCW) wird in einem koronaren Schnitt auf Höhe der hinteren Kommissur gemessen (Normalwert 100–120 Grad) und beträgt bei Patienten mit iNPH zwischen 50–80 Grad (< 90 Grad; [1]).

Bei kompletter Hakim-Symptomtrias und bildgebenden Hinweisen auf einen iNPH ist die Diagnose wahrscheinlich [1]. Vor Behandlung eines iNPH mit ventrikuloperitonealem Shunt (VP-Shunt) werden ein Liquorablassversuch oder, in weniger eindeutigen Fällen, die lumbale Drainage empfohlen [1, 6]. Der Liquorablassversuch entspricht dem diagnostischen Standard, der die VP-Shuntindikation in positiven Fällen stützt, in negativen Fällen jedoch nicht verwertbar ist [1]. Es werden 30–50 ml Liquor entnommen und Gangparameter wie Schrittlänge, Gehgeschwindigkeit und Wendeschrittzahl vor und nach Liquorablass erfasst, wobei die Verbesserung eines oder mehrerer Parameter einem positiven Prädiktor für das Ansprechen auf einen VP-Shunt entspricht [2, 8]. Verbesserungen im Zeitintervall von 24–48 h nach Liquorablass erlauben die beste Vorhersage [1, 2]. Der positive prädiktive Wert beträgt 90–100 % und der negative prädiktive Wert 30–50 % [6]. Klinische Prädiktoren für ein Ansprechen auf einen Liquorablassversuch sind eine vordergründige Gangstörung, ein Auftreten der Gangstörung vor kognitiven Defiziten sowie eine nur kurze Anamnese hinsichtlich kognitiver Defizite [1]. Als negative Prädiktoren gelten das Vorliegen einer Alzheimer-Demenz mit typischem Liquorprofil, der Nachweis einer schweren kortikalen Atrophie sowie eine ausgeprägte zerebrale Mikroangiopathie [1]. Die lumbale Drainage mit einer Drainagerate von 5–10 ml/h über 2 bis 7 Tage wies in kleineren Fallserien eine Spezifität und Sensitivität vom 100 % für das Ansprechen auf einen VP-Shunt auf [9], wobei mögliche Komplikationen wie eine Meningitis oder Subduralhämatome zu bedenken sind [6], weshalb die lumbale Drainage Fällen mit inkompletter Trias oder negativem Liquorablassversuch vorbehalten sein sollte [1]. Gemäß S1-Leitlinie sollte der Test angewendet werden, mit dem die versorgende Klinik über die größere Erfahrung verfügt [1, 2].

In etwa 77 % der iNPH-Fälle bestehen Komorbiditäten wie eine Alzheimer-Pathologie, ein neurodegenerativ bedingtes Parkinson-Syndrom oder eine relevante zerebrale Mikroangiopathie, wobei insbesondere die Alzheimer-Erkrankung (AD) und die zerebrale Mikroangiopathie im höheren Lebensalter häufig sind und auch eine iNPH-typische Ventrikelerweiterung imitieren können [10, 11]. Insbesondere beim Auftreten kognitiver Defizite vor der Entwicklung einer Gangstörung oder dem Vorhandensein kortikaler Zeichen muss das Vorliegen einer AD in Betracht gezogen werden [12]. Die Daten zur Koinzidenz von iNPH und AD sind variabel, was durch verschiedene Erfassungen der Neuropathologie bedingt ist [13]. Ein diesbezüglich mittlerweile etabliertes Klassifikationssystem zur Beschreibung der Alzheimer-typischen Neuropathologie ist die ATN-Klassifikation (A = Amyloid, T = Tau, N = Neurodegeneration), welche auch in dieser Arbeit Anwendung zur biologischen Charakterisierung der Alzheimer-Erkrankung findet [14]. Eventuelle Verfälschungen der Liquorproteinkonzentrationen durch die iNPH-assoziierte Kompression des Gehirns mit negativen Effekten auf das glymphatische System und verminderter Drainage von Amyloid-Precursor-Proteinfragmenten und Tau-Protein-Spezies mit möglicherweise falsch-niedrigen Werten sind jedoch bei der Liquordiagnostik beim iNPH zu bedenken [15, 16].

Bei einem durchschnittlichen Alter bei Shuntanlage von 75 Jahren verwundert die häufige Koinzidenz von iNPH und AD nicht [4, 17]. Die große Häufigkeit der Koinzidenz beider Erkrankungen lässt jedoch pathophysiologische Überschneidungen vermuten, welche bislang unzureichend verstanden sind [2]. Insbesondere der Einfluss einer komorbiden AD auf Prognose und Therapieansprechen ist Gegenstand aktueller Untersuchungen [2].

Eine Amyloid-PET-Studie bei Patienten mit möglichem iNPH zeigte beispielsweise, dass ein fehlender Amyloidnachweis mit Verbesserung der Gangparameter nach Liquorablass assoziiert ist [18]. Andere Kollegen zeigten hingegen, dass iNPH-Patienten mit Nachweis einer Tau- und Amyloidpathologie mehr als andere vom Liquorablass profitierten [19]. Es ist somit weiterhin unklar, ob der Nachweis von Neurodegenerations- und -destruktionsparametern das kurz- und mittelfristige Ansprechen auf einen Liquorablass beim iNPH beeinflusst und ob die Behandlung eines iNPH mit begleitender AD zumindest in der Frühphase der Erkrankung trotz späterem Überwiegen demenzieller Symptome gerechtfertigt ist [1, 7].

Vor diesem Hintergrund war es das Ziel der vorliegenden Studie, die Koinzidenz von iNPH und AD sowie den Einfluss einer komorbiden AD auf das Ansprechen auf den Liquorablassversuch oder die lumbale Drainage in einem prospektiven Beobachtungsansatz zu analysieren.

## Studiendesign und Untersuchungsmethoden

### Studienteilnehmer

In dieser Beobachtungsstudie analysierten wir alle Patienten, die sich bei klinischem und bildgebendem Verdacht auf einen iNPH im Rahmen der klinischen Routinediagnostik zwischen dem 01.07.2022 und dem 30.06.2023 einer leitliniengerechten NPH-spezifischen Routinediagnostik inklusive Liquorablassversuch oder lumbaler Drainage unterzogen. Jegliche Anhaltspunkte für das Vorliegen eines sekundären Normaldruckhydrozephalus (Vorhandensein eines Hirntumors, Z. n. Enzephalitits/Meningitis, Z. n. Strahlentherapie, Z. n. Bestrahlung, Aquäduktstenose) oder das Vorhandensein eines VP-Shunts waren Ausschlusskriterien, welche mittels Aktensichtung erfasst wurden. Es handelte sich um eine reine Beobachtungsstudie ohne studienspezifische Interventionen. Die klinischen Angaben wurden gemäß klinischer Routine und je nach Einzelfall anhand von Eigen- und Fremdanamnese sowie anhand von Vorepikrisen erhoben. Die Rekrutierung erfolgte aus den Kliniken für Neurologie, Neurochirurgie sowie Psychiatrie und Psychotherapie (akutgeriatrische Station und Universitäts-DemenzCentrum) des Universitätsklinikums Carl Gustav Carus in Dresden. Die kooperierenden Kliniken wurden durch Aushänge und Poster auf diese Beobachtungsstudie aufmerksam gemacht und meldeten entsprechende Fällen den studiendurchführenden Autoren. Das Vorliegen NPH-relevanter MRT- bzw. CT-Parameter wie CCW, Evans-Index und Fazekas-Score wurde durch systematische Analyse der Bilddatensätze erfasst. Mittels Akteneinsicht wurden relevante soziodemografische, klinische, kognitive und liquordiagnostische Parameter erfasst. Gemäß ATN-Klassifikation wurden die lumbalpunktierten Patienten, bei denen im Rahmen der klinischen Routine Demenz- und Destruktionsparameter bestimmt wurden, anhand von Amyloid- und Tau-Parameter-Befunden in A^+^T^+^, A^+^T^−^ und A^−^T^−^ (ohne Erfassung von Neurodegenerationsparametern) kategorisiert. Für das „A“-Kriterium fanden eine pathologisch erniedrigte Aβ-Ratio und ein erniedrigtes Aβ42 Berücksichtigung, während hinsichtlich des „T“-Kriteriums ausschließlich ein erhöhtes Phospho-Tau (pTau) berüchtigt wurde. Ein positives Ethikvotum der lokalen Ethikkommission lag vor (BO-EK-100032022).

### MRT- bzw. CT-Evaluation und NPH-Diagnose

Die cMRT- und cCT-Datensätze wurden durch erfahrene Radiologen (SS, KF) hinsichtlich des Vorliegens und Ausmaßes NPH-spezifischer Marker und hinsichtlich relevanter Kopathologien analysiert. Es erfolgte die Bestimmung von Evans-Index, Corpus-callosum-Winkel und Fazekas-Score. Der Verdacht auf das Vorliegen eines NPH sowie die Beurteilung des Ansprechens erfolgten im Rahmen der klinischen Routine gemäß den in der S1-Leitlinie konsentierten Empfehlungen (iNPH-Verdacht bei positiven klinischen und bildgebenden Kriterien, Ansprechen bei signifikanter Verbesserung der Gangstörung; [1]). Als klinischer Standard für das Ansprechen auf den Liquorablassversuch am Untersuchungsstandort galt, in Übereinstimmung mit den konsentierten leitlinienbasierten Empfehlungen [1], eine 20 %ige Verbesserung der Ganggeschwindigkeit in einem standardisierten 10-m-Gehstreckentest nach Liquorablass.

### Statistische Auswertung

Demografische und klinische Charakteristika wurden mit Mittelwerten und Standardabweichungen (SD) für kontinuierliche Variablen und mittels absoluter und relativer Häufigkeiten für kategoriale Variablen beschrieben. In Abhängigkeit von Datenniveau und -verteilung wurden t‑Tests, χ^2^-Tests, Varianzanalysen (ANOVAs) und logistische Regressionsanalysen durchgeführt. Insgesamt wurden drei logistische Regressionen gerechnet. Unabhängige Variablen waren in der ersten Regressionsanalyse die Symptomdauer, in der zweiten der MoCA sowie der MMST, beide vor Liquorablass, und in der dritten Analyse der CCW in Grad. Es wurde jeweils der Einfluss der unabhängigen Variablen auf das Ansprechen im Liquorablass (abhängige Variable) untersucht (verwendete Abkürzungen: B = Regressionskoeffizient, SF = Standardfehler, df = Freiheitsgrade, Exp[B] = Odds Ratio). Die statistische Auswertung erfolgte mit dem Programm IBM SPSS Statistics für Windows, Version 29.

## Ergebnisse

### Klinisches und kognitives Assessment

**Abb. 1:**
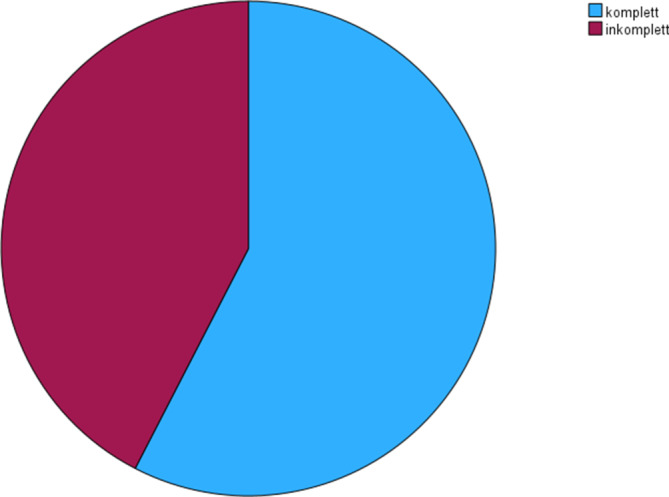
Verteilung komplette vs. inkomplette Hakim-Trias

**Abb. 2:**
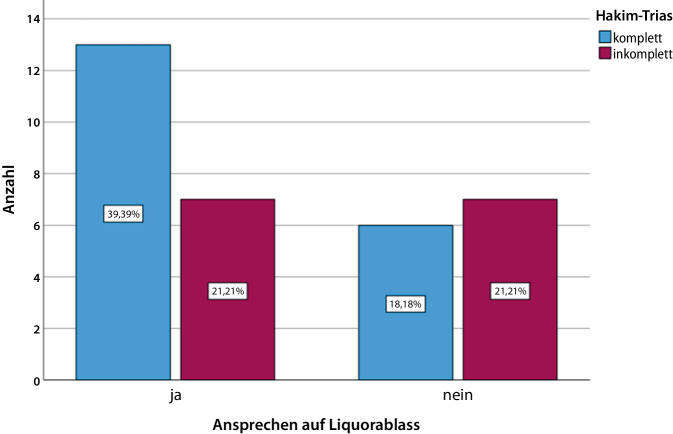
Ansprechen auf Liquorablass in Abhängigkeit von kompletter vs. inkompletter Hakim-Trias

**Abb. 3:**
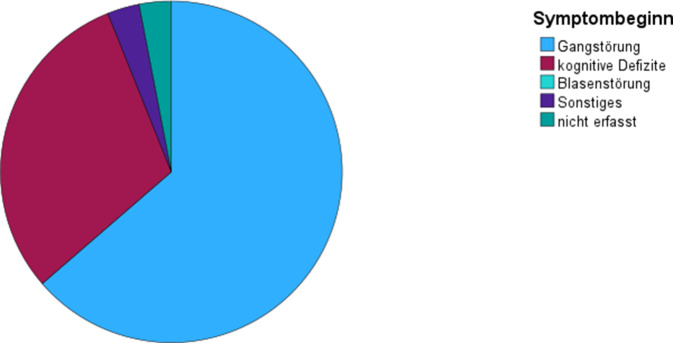
Verteilung Art des 1. Symptoms

Im Beobachtungszeitraum wurden 33 Patienten (14 weiblich, 19 männlich) mit klinischem und bildgebendem Verdacht auf einen iNPH eingeschlossen. Das Durchschnittsalter betrug 74,6 ± 8,1 (54–85) Jahre. 19 Patienten (57,6 %) wiesen eine komplette und 14 Patienten (42,4 %) eine inkomplette Hakim-Trias auf (Abb. 1). Bei 65,0 % der Patienten, die auf den Liquorablass ansprachen, war die Hakim-Trias komplett und bei den Patienten, die nicht ansprachen, war die Hakim-Trias lediglich bei 46,2 % komplett, wobei der Unterschied nicht signifikant war (χ^2^ = 1,146; df = 1; *p* = 0,284; Abb. 2, Tab. 1). Bei 21 Patienten (63,6 %) war eine Gangstörung das erste Symptom und bei 10 Patienten (30,3 %) traten primär kognitive Defizite auf. Ein Patient benannte ein anderes Erstsymptom als Gangstörung, kognitive Defizite oder eine Blasenstörung und bei einem weiteren Patienten wurde das erste Symptom nicht erfasst (Abb. 3). Von den Patienten mit einer primären Gangstörung sprachen 66,7 % auf den Liquorablass an und von den Patienten mit primären kognitiven Defiziten sprachen 40,0 % an. Der Zusammenhang zwischen Ansprechen und Art des ersten Symptoms war nicht signifikant (χ^2^ = 3,402; df = 3; *p* = 0,334), wobei die Fallzahlen sehr klein waren. Bei den Patienten, die auf den Liquorablass ansprachen, betrug die Symptomdauer 25,2 ± 18,5 Monate und bei denen, die nicht ansprachen 32,7 ± 22,9 Monate. Der Einfluss der Symptomdauer auf das Ansprechen war dabei ebenfalls nicht signifikant (B = 0,019; SF = 0,018; df = 1; Exp(B) = 1,019; *p* = 0,303).

**Tab. 1:** Kreuztabelle Ansprechen auf Liquorablass und Hakim-Trias komplett/inkomplett

			Hakim-Trias		Gesamt
			Komplett	Inkomplett	
Ansprechen auf Liquorablass	Ja	Anzahl	13	7	20
		% von Ansprechen auf Liquorablass	65,0 %	35,0 %	100,0 %
	Nein	Anzahl	6	7	13
		% von Ansprechen auf Liquorablass	46,2 %	53,8 %	100,0 %
Gesamt		Anzahl	19	14	33
		% von Ansprechen auf Liquorablass	57,6 %	42,4 %	100,0 %

Insgesamt 19 Patienten erhielten vor Liquorablass einen MMST (MW 23,2 ± SD 6,2) und 30 Patienten einen MoCA (MW 17,3 ± SD 6,5). Insgesamt erhielten zudem 8 Patienten einen MMST nach Liquorablass (MW 24,1 ± SD 6,4) und 25 Patienten einen MoCA nach Liquorablass (MW 18,3 ± SD 6,3). 8 Patienten erhielten vor Liquorablass ein Assessment mittels Frontal Assessment Battery (FAB‑D; MW 11,0 ± SD 4,8) und 7 nach Liquorablass (MW 11,0 ± SD 4,8; [20]). 8 Patienten hatten einen MMST vor und nach Liquorablass (MMST vor MW 23,4 ± SD 6,3; MMST nach MW 24,1 ± SD 6,3; gepaarte Stichprobe) und 25 Patienten einen MoCA vor und nach Liquorablass (MoCA vor MW 17,5 ± SD 6,5; MoCA nach MW 18,3 ± 6,3; gepaarte Stichprobe). Die Unterschiede zwischen dem MMST vor und nach Liquorablass (T = −0,767; df = 7; *p* = 0,468) und dem MoCA vor und nach Liquorablass (T = −1,335; df = 24; *p* = 0,195) waren nicht signifikant (Tab. 2 und 3).

**Tab. 2:** MMST und MoCA-Werte der gepaarten Stichprobenvor und nach Liquorablass

		Mittelwert	*N*	Std.-Abweichung	Standardfehler des Mittelwertes
Paaren 1	MMST vor	23,33	8	6,323	2,236
	MMST nach	24,13	8	6,357	2,243
Paaren 2	MoCA vor	17,52	25	6,520	1,304
	MoCA nach	13,28	25	6,255	1,251

**Tab. 3:** MMST- und MoCA-Differenzen vor und nach Liquorablass

		Mittelwert	Std.-Abweichung	Gepaarte Differenzen			T	Df	Signifikanz	
				Standardfehler Mittelwertes	95 %-Konfidenzintervall der Differenz				Einseitiges *p*	Zweiseitiges *p*
					Unterer Wert	Oberer Wert				
Paaren 1	MMST vor – MMST nach	−0,750	2,765	0,977	−3,061	1,561	−0,767	7	0,234	0,468
Paaren 2	MoCA vor – MoCA nach	−0,760	2,847	0,569	−1,935	0,415	−1,335	24	0,097	0,195

Bei den Patienten, die auf den Liquorablass ansprachen, betrug der MoCA vor Liquorablass 18,3 ± 5,8 (*n* = 17) und der MMST vor Liquorablass 24,8 ± 4,1 (*n* = 12). Bei den Patienten, die nicht ansprachen, lag der MoCA vor Liquorablass bei 15,9 ± 7,4 (*n* = 13) und der MMST bei 20,3 ± 8,3 (*n* = 7). Der Einfluss des MoCA vor Liquorablass (B = 0,118; SF = 0,181; df = 1; Exp[B] = 1,125; *p* = 0,514) und der Einfluss des MMST vor Liquorablass (B = −0,264; SF = 0,232; df = 1; Exp[B] = 0,768; *p* = 0,255) auf das Ansprechen waren jedoch nicht signifikant.

Die Differenz vom MoCA vor und nach Liquorablass betrug bei den Patienten, die ansprachen, im Mittel 1,7 Punkte, während sie bei den Patienten, die nicht ansprachen, bei −0,7 Punkten lag (ANCOVA mit MOCA vor als Kovariate: F[1;22] = 5,725; *p* = 0,026). Die Differenz im MMST vor und nach Liquorablass betrug bei den Patienten, die ansprachen, 1,5 Punkte, bei denen, die nicht ansprachen, −1,5 Punkte (ANCOVA aufgrund zu geringer Fallzahlen nicht gerechnet).

### Bildgebung

Bei 32 Patienten (97,0 %) war der Corpus-callosum-Winkel (CCW) pathologisch (< 90°) und bei 31 Patienten (93,9 %) war der Evans-Index pathologisch (> 0,3). 56,3 % der Patienten mit pathologischem CCW wiesen eine komplette Hakim-Trias auf und 43,8 % eine inkomplette. Bei 17 Patienten (51,5 %) waren die Hakim-Trias komplett und sowohl CCW als auch Evans-Index pathologisch. 62,5 % der Patienten mit pathologischem CCW (*n* = 20) sprachen auf den Liquorablass an und 37,5 % der Patienten mit pathologischem CCW (*n* = 12) nicht. 64,5 % der Patienten mit pathologischem Evans-Index (*n* = 20) sprachen auf den Liquorablass an und 35,5 % der Patienten (*n* = 11) nicht. Von den Patienten mit auffälligem CCW und Evans-Index sprachen hingegen 66,7 % (*n* = 20) an. Dieser Zusammenhang war nicht signifikant, es lag aber ein Trend vor, dass die Patienten mit auffälligem CCW und Evans-Index häufiger ansprechen (Exakter Test nach Fisher, *p* = 0,052). Von den Patienten mit kompletter Hakim-Trias, auffälligem CCW und Evans-Index sprachen sogar 76,5 % auf den Liquorablass an, von den restlichen Patienten waren es lediglich 43,8 %. Auch dieser Zusammenhang war nicht signifikant (χ^2^ = 3,696; df = 1; *p* = 0,055), es lag aber ebenfalls ein Trend vor, dass Patienten mit kompletter Trias, auffälligem CCW und Evans-Index häufiger auf den Liquorablass ansprechen.

Der CCW in Grad lag bei den Patienten, die auf den Liquorablass ansprachen, bei 60,4 ± 14,1 Grad, während der CCW bei denen, die nicht ansprachen, bei 66,7 ± 15,7 Grad lag. Der Einfluss des CCW in Grad auf das Ansprechen auf den Liquorablass war allerdings nicht signifikant (B = 0,031; SF = 0,026; df = 1; Exp[B] = 1,031; *p* = 0,242).

**Abb. 4:**
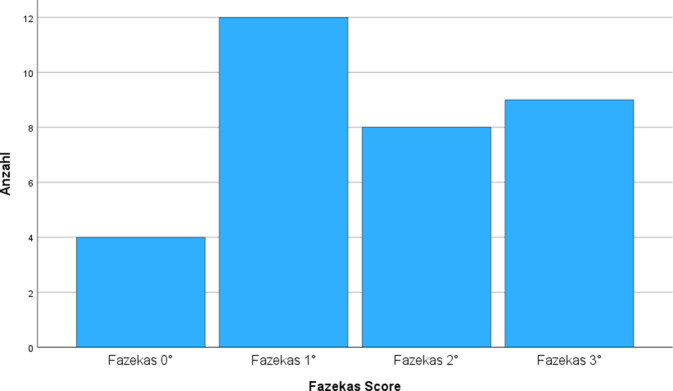
Verteilung der Fazekas-Grade in der Gesamtkohorte

**Abb. 5:**
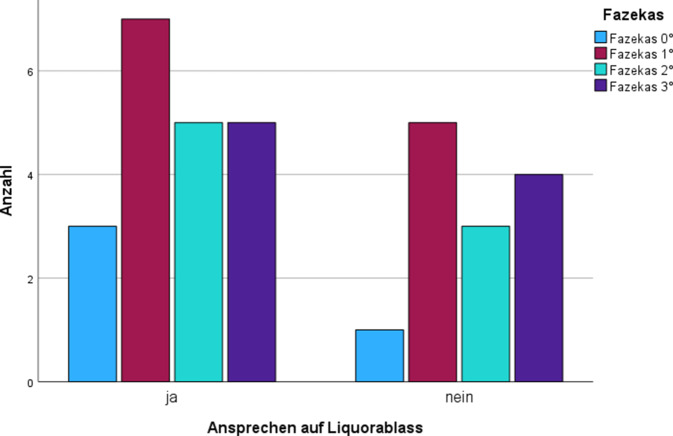
Ansprechen auf Liquorablass in Abhängigkeit vom Fazekas-Score

Der durchschnittliche Fazekas-Score in der Gesamtkohorte betrug 1,7 ± 1,0. Eine Übersicht zur Verteilung der Fazekas-Grade findet sich in Abb. 4. Bei den Patienten, die auf den Liquorablass ansprachen, betrug der durchschnittliche Fazekas-Score 1,6 ± 1,0 und bei denen, die nicht ansprachen 1,8 ± 1,0. Von den Patienten mit Fazekas-Score 0 (*n* = 4) sprachen 75,0 % (*n* = 3) auf den Liquorablass an, von den Patienten mit Fazekas-Score 1 (*n* = 12) 58,3 % (*n* = 7), von den Patienten mit Fazekas-Score 2 (*n* = 8) 62,5 % (*n* = 5) und von denen mit Fazekas-Score 3 (*n* = 9) 55,6 % (*n* = 5; Abb. 5). Von allen Patienten mit Fazekas-Score > 0 (*n* = 29) sprachen insgesamt 58,6 % (*n* = 17) auf den Liquorablass an (Tab. 4). Dabei bestand kein Zusammenhang zwischen Fazekas-Score und Ansprechen auf den Liquorablass (Fazekas-Score 0 vs. Fazekas-Score > 0: Exakter Test nach Fisher: *p* = 0,481; Fazekas-Score 0 und 1 vs. Fazekas-Score > 1: Exakter Test nach Fisher: *p* = 0,556).

**Tab. 4:** Kreuztabelle Fazekas-Score und Ansprechen auf Liquorablass

			Ansprechen auf Liquorablass		Gesamt
			Ja	Nein	
Fazekas	Fazekas 0°	Anzahl	3	1	4
		% von Fazekas	75,0 %	25,0 %	100,0 %
	Fazekas 1°	Anzahl	7	5	12
		% von Fazekas	53,3 %	41,7 %	100,0 %
	Fazekas 2°	Anzahl	5	3	8
		% von Fazekas	62,5 %	37,5 %	100,0 %
	Fazekas 3°	Anzahl	5	4	9
		% von Fazekas	55,6 %	44,4 %	100,0 %
Gesamt		Anzahl	20	13	33
		% von Fazekas	60,6 %	39,4 %	100,0 %

### Liquordiagnostik

**Abb. 6:**
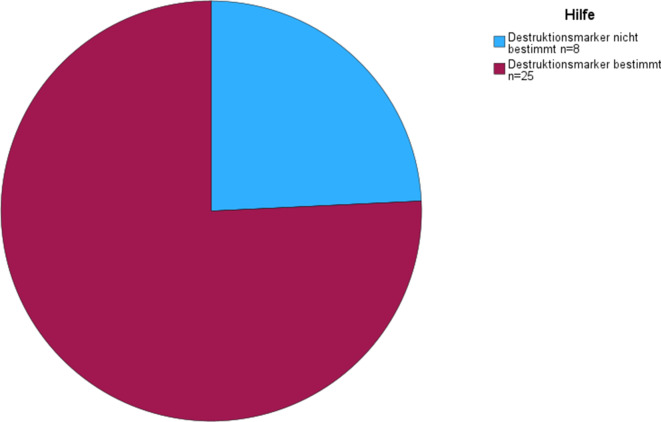
Übersicht zur Häufigkeit der Bestimmung von Demenz- und Destruktionsmarkern

**Abb. 7:**
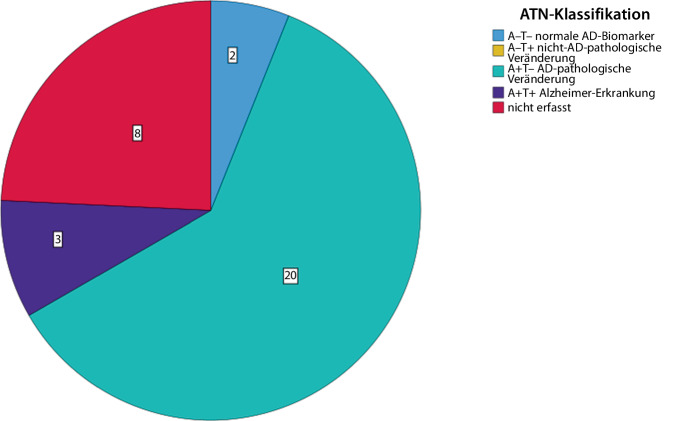
Einteilung gemäß ATN-Klassifikation. *AD* Alzheimer-Erkrankung

Insgesamt erhielten 15 Patienten (45,5 %) eine Lumbalpunktion und 18 eine lumbale Drainage (54,5 %). Bei Patienten mit kompletter Hakim-Trias erhielten 9 (47,4 %) eine Lumbalpunktion und 10 (52,6 %) eine lumbale Drainage. Von den Patienten mit inkompletter Hakim-Trias erhielten 6 (42,9 %) eine Lumbalpunktion und 8 (57,1 %) eine lumbale Drainage. Die Bestimmung des Liquoreröffnungsdruckes erfolgte lediglich bei 7 Patienten (20,43 cmH_2_0 ± 2,07, 18–23 cmH_2_0). Bei 25 (75,8 %) Patienten erfolgte die begleitende Bestimmung von Demenz- und Destruktionsparametern (Abb. 6). Der MMST bei Patienten mit erfolgter Bestimmung von Demenz- und Destruktionsmarkern (*n* = 19) lag vor Liquorablass bei 23,2 ± 6,2 und der MoCA bei 17,2 ± 6,9 (*n* = 24). Von den Patienten mit bestimmten Demenz- und Destruktionsmarkern fielen 20 (80,0 %) in die Kategorie A^+^T^−^, 3 (12,0 %) in die Kategorie A^+^T^+^ und 2 (8,0 %) in die Kategorie A^−^T^−^ (Abb. 7). Bei 66 % der Patienten der A^+^T^+^-Kategorie und bei 30 % der A^+^T^−^-Patienten fiel eine pathologisch erniedrigte Aβ-Ratio auf. Bei den 3 Patienten mit positiven Amyloid- und Tau-Parametern (A^+^T^+^) betrug der MMST vor Liquorablass 15,5 ± 9,2 (*n* = 2) und der MoCA 10,7 ± 7,6 (*n* = 3). Der MMST nach Liquorablass betrug in dieser Gruppe (A^+^T^+^) 16,5 ± 9,2 und der MoCA 10,5 ± 9,2. In der A^−^T^−^-Gruppe betrug der MMST vor Liquorablass 26,0 ± 1,4 (*n* = 2) und der MoCA 20,0 ± 4,2 (*n* = 2). Nach Liquorablass lag der MMST in der A^−^T^−^-Gruppe bei 21,0 (*n* = 1) und der MoCA bei 17,5 ± 2,1 (*n* = 2). Von den 2 A^−^T^−^-Patienten sprach ein Patient auf den Liquorablass an (50 %), von den 3 A^+^T^+^-Patienten sprach ebenfalls ein Patient (33,3 %) und von allen A^+^T^+^- und A^+^T^−^-Patienten sprachen 15 Patienten (65,2 %) an. A^+^T^+^- und A^+^T^−^-Patienten sprachen jedoch nicht signifikant häufiger an als A^−^T^−^-Patienten (Exakter Test nach Fisher; *p* = 0,600). Und auch die A^+^T^−^-Patienten sprachen nicht signifikant häufiger auf den Liquorablass an als A^−^T^−^-Patienten (Exakter Test nach Fisher; *p* = 0,545).

Bei den A^+^T^−^-Patienten betrug der MMST vor Liquorablass 23,8 ± 5,8 und der MoCA 17,9 ± 6,7. Bei der Gruppe aller A^+^T^+^- und A^+^T^−^-Patienten betrug der MMST vor Liquorablass 22,8 ± 6,5 und danach 24,6 ± 6,7; der MoCA in dieser Gruppe betrug 16,9 ± 7,1 vor und 17,8 ± 7,2 nach Liquorablass. Aufgrund der geringen Fallzahl in der A^−^T^−^- sowie in der A^+^T^+^-Gruppe waren statistische Tests nicht sinnvoll. In der Varianzanalyse ergaben sich keine signifikanten Zusammenhänge zwischen den einzelnen Proteinkonzentrationen und dem Ansprechen auf den Liquorablassversuch (Aβ42: F[1;23] = 0,016, *p* = 0,900; Aβ-Ratio: F[1;23]=0,006, *p* = 0,941; pTau: F[1;23] = 0,652, *p* = 0,428; Tau: F[1;23] = 1,055, *p* = 0,315).

## Diskussion

Die Ergebnisse dieser Beobachtungsstudie belegen zunächst die große klinische Heterogenität von Patienten mit klinischem und bildgebendem iNPH-Verdacht, deren umfängliche Erfassung inklusive relevanter Komorbiditäten in der prognostischen Abschätzung des Ansprechens auf einen Liquorablass und somit zur Vermeidung unnötiger operativer Prozeduren von größter klinischer Bedeutung ist. In Übereinstimmung mit der verfügbaren Literatur wiesen in der untersuchten Kohorte beispielsweise weniger als 60 % der Patienten eine komplette Hakim-Trias auf (57,6 %; [4, 5]) und lediglich bei 63,6 % war die Gangstörung das erste Symptom. Auch die Altersstruktur der untersuchten Kohorte mit einem Durchschnittsalter von 74,6 Jahren belegt, dass es sich, in Anbetracht eines gemäß aktueller Literatur durchschnittlichen Alters von 75 Jahren bei Shunt-Anlage [4, 17], um eine repräsentative klinische Stichprobe handelt.

Passend zu den leitlinienbasierten Empfehlungen zum kognitiven Assessment im Rahmen der NPH-Diagnostik fand mehrheitlich der MoCA-Test Anwendung [1]. In Ergänzung zur Leitlinie wurde bei 8/33 Patienten zusätzlich die zur Detektion frontosubkortikaler Defizite geeignetere Frontal Assessment Battery (FAB) eingesetzt [20]. Annehmbar aufgrund der kleinen Fallzahl konnte weder für den MMST- und MoCA-Testwert noch für die Symptomdauer ein signifikanter Einfluss auf das Ansprechen auf den Liquorablass nachgewiesen werden. Allerdings bestand ein signifikanter Zusammenhang zwischen größerer Differenz der MoCA-Punktwerte vor und nach Liquorablass und Ansprechen auf den Liquorablass.

Mit einem durchschnittlichen Fazekas-Score von 1,7 in der untersuchten Kohorte demonstrieren die Ergebnisse dieser Studie darüber hinaus, dass insbesondere eine zerebrovaskuläre Kopathologie bei iNPH-Patienten häufig ist, wenngleich in der untersuchten Kohorte kein Einfluss der zerebralen Mikroangiopathie auf das Ansprechen im Liquorablass nachweisbar war. Bezüglich der weiteren bildgebenden Befunde deuten die Ergebnisse dieser Analyse außerdem darauf hin, dass die standardisierte Erfassung von CCW und Evans-Index und insbesondere deren kombinierte Erhebung und Kontextualisierung mit klinischen Befunden tendenzielle Vorteile in der prognostischen Abschätzung des Ansprechens auf einen Liquorablass bietet. So bestand ein Trend, dass Patienten mit auffälligem CCW und Evans-Index häufiger ansprechen (*p* = 0,052). Darüber hinaus bestand auch ein Trend, dass Patienten mit kompletter Hakim-Trias, auffälligem CCW und Evans-Index häufiger auf den Liquorablass ansprechen.

Ein wesentliches Ergebnis dieser Analyse ist die große Häufigkeit einer komorbiden Amyloidpathologie bei Patienten mit iNPH-Verdacht. Gemäß biologischer Definition der Alzheimer-Erkrankung bedarf es für den Nachweis einer Alzheimer-Erkrankung auffälliger Amyloid- und Tau-Parameter (Aβ42-Erniedrigung bzw. pathologisch erniedrigte Aβ-Ratio sowie erhöhtes pTau-Protein im Liquor; [14, 21]), wobei neueste diagnostische Klassifikationssysteme bereits den alleinigen Nachweis einer Amyloidproteinopathie mit der Diagnose einer Alzheimer-Erkrankung gleichsetzen [22]. Insgesamt erfolgte bei 75,8 % der Patienten die Bestimmung von Demenz- und Destruktionsparametern. In der vorliegenden Analyse wiesen insgesamt 92 % der Patienten auffällige Amyloidparameter auf (A^+^T^+^ und A^+^T^−^), wenngleich lediglich bei 12 % der Patienten auffällige Amyloid- und Tau-Parameter (A^+^T^+^) nachgewiesen wurden.

Auch wenn die Mehrzahl der amyloidpositiven Patienten isoliert auffällige Amyloidparameter zeigte, ist gemäß aktueller Studienergebnisse von einer sehr hohen Rate an Amyloidkopathologien auszugehen. Aktuelle Längsschnittuntersuchungen inklusive Post-mortem-Analysen zeigen, dass bei A^+^T^+^-Patienten in 100 % der Fälle eine Alzheimer-Erkrankung zugrunde liegt, während dies bei A^+^T^−^-Patienten (ohne iNPH) noch in 50–73 % der Fälle zutrifft [23]. In diesem Zusammenhang ist jedoch darauf zu verweisen, dass im Rahmen des iNPH eine verminderte Clearance Alzheimer-spezifischer Proteine angenommen wird, sodass die hohe Rate an Amyloidkopathologie in der untersuchten Kohorte gegebenenfalls einer Relativierung bedarf, wobei die aktuelle Datenlage diesbezüglich nicht ausreichend ist, um eventuelle Clearance-Effekte auf Liquorproteinkonzentrationen bei Patienten mit iNPH quantitativ abschätzen zu können. So zeigen aktuelle metaanalytische Daten zwar verminderte Tau- und pTau-Proteinkonzentrationen im Liquor von iNPH-Patienten im Vergleich zu Patienten mit einer Alzheimer-Erkrankung, wobei die Aβ42-Protein-Konzentrationen im Liquor bei Patienten mit iNPH in diesen Analysen signifikant über denen von Patienten mit Alzheimer-Erkrankung lagen, sodass die Effekte des iNPH auf Aβ42-Konzentrationen im Liquor zum aktuellen Zeitpunkt auch nicht überschätzt werden dürfen [24]. Nichtsdestotrotz zeigen diese metaanalytischen Daten auch, dass iNPH-Patienten signifikant geringere Aβ42-Konzentrationen im Liquor aufweisen als gesunde Kontrollen, sodass ein gewisser Einfluss des iNPH auf die Aβ42-Konzentration im Liquor auch keinesfalls ausgeschlossen werden kann. Vor diesem Hintergrund ist in der untersuchten Kohorte dennoch von einer beträchtlichen, wenngleich auch nicht exakt zu quantifizierenden Rate komorbider Alzheimer-Erkrankungen auszugehen.

Zu den unmittelbaren, querschnittlichen Effekten einer zum iNPH komorbiden Aymloidpathologie auf das Ansprechen im Liquorablass existieren uneinheitliche Daten. Während Pyrgelis und Kollegen demonstrieren, dass pathologische Amyloid- und Tau-Parameter mit einem schlechteren Ansprechen von iNPH-Patienten im Liquorablass assoziiert sind, zeigen Müller-Schmitz et al. gegenteilige Effekte einer komorbiden Alzheimer-Erkrankung im Sinne eines vergleichsweise besseren Ansprechens [19, 25]. Darüber hinaus berichten Gold und Kollegen keinen signifikanten Einfluss einer komorbiden Alzheimer-Erkrankung auf das querschnittliche Ansprechen, wenngleich längsschnittlich größere kognitive Abbauraten, insbesondere in exekutiven Domänen, bei Patienten mit komorbider Alzheimer-Erkrankung im Vergleich zum isolierten iNPH beschrieben werden [26]. Passend zu den Befunden von Gold et al. bildeten sich auch in der untersuchten Kohorte keine signifikanten Effekte einer komorbiden Amyloidpathologie auf das unmittelbare Ansprechen im Liquorablass ab.

Zusammenfassend demonstrieren die Ergebnisse dieser prospektiven Beobachtungsstudie die klinische Heterogenität von iNPH-Patienten, den zusätzlichen und dem MMST überlegenen Wert von Differenzen im MoCA-Punktwert vor und nach Liquorablass in der Abbildung kognitiver Verbesserungen von Patienten, die auf einen Liquorablass motorisch respondieren, den zusätzlichen Wert der kombinierten Erhebung von CCW und Evans-Index und deren Kontextualisierung mit klinischen Befunden in der prognostischen Abschätzung des Ansprechens auf den Liquorablassversuch, die große Häufigkeit einer komorbiden Amyloidpathologie bzw. AD sowie einer vaskulären Kopathologie bei Patienten mit klinischem und bildgebendem iNPH-Verdacht. Ein Einfluss einer komorbiden AD auf das Ansprechen im Liquorablassversuch konnte nicht nachgewiesen werden.

Die wesentliche Stärke dieser Studie liegt darin, dass es sich um eine repräsentative klinische Stichprobe aus dem praktischen Versorgungsalltag handelt. Insbesondere bei der Beschreibung der Effekte einer komorbiden Amyloidpathologie auf das Ansprechen im Liquorablassversuch muss jedoch die geringe Fallzahl als wesentliche Limitation dieser Studie berücksichtigt werden.

## Fazit für die Praxis

- Bei der Abbildung kognitiver Verbesserungen bei Patienten, die auf einen Liquorablassversuch respondieren, sind Differenzen im MoCA-Test denen im MMST überlegen.
- Die kombinierte Bestimmung von CCW und Evans-Index sowie deren Kontextualisierung mit klinischen Charakteristika verbessern möglicherweise die prognostische Abschätzung eines Ansprechens auf einen Liquorablassversuch.
- Eine vaskuläre Kopathologie in Form einer zerebralen Mikroangiopathie ist bei iNPH-Patienten häufig.
- Eine komorbide Amyloidpathologie bzw. Alzheimer-Erkrankung ist bei iNPH-Patienten sehr häufig, wobei deren exakte Häufigkeit aufgrund anzunehmender iNPH-Effekte auf die Clearance Alzheimer-typischer Liquorproteine nicht genau quantifiziert werden kann.
- Der Einfluss einer komorbiden Amyloidpathologie auf das unmittelbare Ansprechen im Liquorablassversuch ist aktuell noch ungeklärt.

## Data Availability

Die in dieser Studie erhobenen Datensätze können auf begründete Anfrage beim Korrespondenzautor angefordert werden.
